# Access to hospital and community palliative care for patients with advanced cancer: A longitudinal population analysis

**DOI:** 10.1371/journal.pone.0200071

**Published:** 2018-08-08

**Authors:** Cheryl L. Craigs, Robert M. West, Adam Hurlow, Michael I. Bennett, Lucy E. Ziegler

**Affiliations:** 1 St Gemma’s Academic Unit of Palliative Care, Leeds Institute of Health Sciences, Level 10, Clarendon Way, University of Leeds, Leeds, United Kingdom; 2 Health Services Research, Leeds Institute of Health Sciences, Level 11, Clarendon Way, University of Leeds, Leeds, United Kingdom; 3 Palliative Care Team, St James’s University Hospital, Leeds, United Kingdom; National Cancer Institute, UNITED STATES

## Abstract

**Background:**

The UK National Health Service is striving to improve access to palliative care for patients with advanced cancer however limited information exists on the level of palliative care support currently provided in the UK. We aimed to establish the duration and intensity of palliative care received by patients with advanced cancer and identify which cancer patients are missing out.

**Methods:**

Retrospective cancer registry, primary care and secondary care data were obtained and linked for 2474 patients who died of cancer between 2010 and 2012 within a large metropolitan UK city. Associations between the type, duration, and amount of palliative care by demographic characteristics, cancer type, and therapies received were assessed using Chi-squared, Mann-Whitney or Kruskal-Wallis tests. Multinomial multivariate logistic regression was used to assess the odds of receiving community and/or hospital palliative care compared to no palliative care by demographic characteristics, cancer type, and therapies received.

**Results:**

Overall 64.6% of patients received palliative care. The average palliative care input was two contacts over six weeks. Community palliative care was associated with more palliative care events (p<0.001) for a longer duration (p<0.001). Patients were less likely to receive palliative care if they were: male (p = 0.002), aged 80 years or over (p<0.05), diagnosed with lung cancer (p<0.05), had not received an opioid prescription (p<0.001), or had not received chemotherapy (p<0.001). Patients given radiotherapy were more likely to receive community only palliative care compared to no palliative care (Odds Ratio = 1.49, 95% Confidence Interval = 1.16–1.90).

**Conclusion:**

Timely supportive care for cancer patients is advocated but these results suggest that older patients and those who do not receive anti-cancer treatment or opioid analgesics miss out. These patients should be targeted for assessment to identify unmet needs which could benefit from palliative care input.

## Background

For patients with advanced cancer, access to palliative care can improve quality of life and reduce hospitalisations, aggressive anti-cancer treatment and the chance of dying in hospital [1–7]. The research evidence to support this is mainly derived from studies undertaken in North America and is beginning to influence policy within the US [8]. Within the UK, research in this area has been slower to progress and there is currently no evidence to draw on from a UK National Health Service context about what represents usual care in relation to access to palliative care for patients with advanced cancer. A key challenge is the UK model of palliative care delivery has evolved to be inherently diverse with patients receiving palliative care across various settings ranging from the patient’s own home to outpatient clinics, inpatient wards, hospices, acute hospitals and nursing homes. This makes it difficult to establish the proportion of cancer patients receiving palliative care in these contexts and the typical duration of care. The US research evidence also leaves questions unanswered. It is unclear which palliative care interventions led to improvements in outcomes, which patients would benefit most and when in the course of disease, integration of palliative care should occur [9]. Despite these challenges, in July 2016 an Enhanced Supportive Care (ESC) initiative was launched in 21 cancer centres across England underpinned by the US research evidence [1–7] and supported by incentives from NHS England [10].

This study aims to establish the proportion of cancer patients receiving community or hospital based palliative care before death, describe the duration and intensity of the palliative care provided, and the associations between palliative care provision and patient characteristics. This will enable us to benchmark local practice against the US research evidence which is driving the ESC initiative, provide a baseline against which to measure its impact and determine which cancer patients are currently underrepresented, in terms of access to palliative care, to help target ESC towards those who may benefit most.

## Methods

### Design

A retrospective analysis of deceased cancer patient data obtained from registry, primary care and secondary care sources. An open Pseudonymiser system was used to link datasets, using an encrypted code based on NHS numbers. Registry data was obtained from the Northern and Yorkshire Cancer Registry (NYCR). Primary care data was obtained from SystmOne, a UK wide medical record system used by approximately three quarters of GP practices in Leeds. The Patient Pathway Manager (PPM), a clinical information system used at Leeds Teaching Hospitals Trust (LTHT) to manage and coordinate care, provided secondary care data.

Ethical approval for the study was granted by the National Research Ethics Service (PR 13.YH.0301).

### Participants

Deceased patients, registered on the NYCR and SystmOne who died of cancer between January 2010 and February 2012 and were at least 18 years of age at death were selected for inclusion.

### Measures

#### Palliative care

Community palliative care provision was calculated using GP communications within SystmOne. In the first stage, all SystmOne records for eligible patients were extracted if they included a palliative care based Read code, or included text indicating palliative care had been received. Read codes are used within primary care in the UK as a standard tool to record patient records, findings, and procedures [11]. In the second stage community palliative care provision was identified as having taken place if the GP record referred to communication with a hospice, or if the Read codes indicated active palliative care had taken place. The decision as to whether a Read code indicated active palliative care was identified through consensus between the authors CLC, MIB, and LEZ. The list of all READ codes identified, with READ codes indicating active palliative care, are provided in S1 Table. Each unique date in which palliative care provision was recorded within SystmOne was identified as a unique community palliative care event.

Hospital palliative care provision was provided by PPM from their palliative care referral database. Each unique palliative care referral date recorded on the PPM system was identified as a unique hospital palliative care event.

Three indicators were used to define palliative care provision. The first indicator identified if patients received palliative care and, if so, the provider of this care (community or hospital). The remaining indicators represented the duration and intensity of palliative care received. These were calculated using the difference (in weeks) between date of the first palliative care event and date of death, and the number of palliative care events, respectively.

#### Demographic characteristics

NYCR provided the demographic information age at death, sex, and deprivation. Deprivation was measured using the Indices of Multiple Deprivation (IMD) quintiles (1 most deprived; 5 least deprived); a measure of neighbourhood deprivation based on income, employment, education, health, crime, access to services, and living environment. Hospital admissions to LTHT between diagnosis and death were obtained from the PPM.

#### Cancer characteristics

Cancer diagnosis and cancer diagnosis date were provided by NYCR. The first cancer diagnosis was used for multiple cancer diagnoses. Duration of illness, was calculated by subtracting week of death from week of diagnosis.

#### Therapies received

SystmOne provided information on analgesic prescriptions within the last year of life. Chemotherapy and radiotherapy data were provided by PPM.

### Analysis

Frequencies and percentages were used to describe categorical data. The median and inter quartile range were used to describe continuous data. The likelihood of independence between categorical variables was assessed using the Pearson’s chi-squared test (χ^2^). Comparisons of continuous data between categories were assessed using the Mann-Whitney test (two categories) or the Kruskal-Wallis test (three or more categories).

A multinomial multivariable logistic regression model was used to investigate the odds of receiving community, hospital, or both palliative care, compared to no palliative care, taking into account all demographic characteristics, cancer characteristics and therapies received. Results are presented as odds ratios (ORs) alongside 95% confidence intervals. Subgroup analysis was performed for the multinomial multivariable logistic regression by duration of illness, with patients analysed separately based on the following groups: survival under 6 months, survival 6 months to two years, and survival over two years.

Only patients with complete demographic and prescription information were included in the analysis. This reduced the cohort from 2479 to 2474 patients (Missing: IMD = 4, missing opioid information = 1).

## Results

### Demographic characteristics

Table 1 shows comparisons between demographic patient characteristics and palliative care provision. Of the 2474 patients included in the study 1597 (64.6%) received palliative care, with a median of two contacts over a 6 week duration before death. Community palliative care was received by 1124 patients (45.4%) and hospital palliative care was received by 990 patients (40.0%). Approximately one in five (517/2474) patients received both community and hospital palliative care. Patients received more palliative care events (p<0.001), for a longer duration (p<0.001), in the community compared to hospital. Patients who received hospital palliative care generally only received one contact. The likelihood of receiving palliative care, and the type of palliative care received, was not significantly related to having had a hospital admission.

**Table 1:** Demographic characteristics by palliative care provision.

	Total (No.)	Palliative care received (Row %)				None vs. Any p value^a^	Sub-group receiving palliative care (n = 1597)					
		None	Community only	Hospital only	Community & Hospital		Palliative care events			Palliative care duration (weeks)		
							Median	IQR	Mean Rank	Median	IQR	Mean Rank
**Total number of participants**												
	2474	35.4	24.5	19.1	20.9	n/a	2	1 to 3		6	2 to 19	
**Palliative care provider**												
Community only	607						2 (i,ii)	1 to 3	767.9	7 (i,ii)	2 to 24	824.1
Hospital only	473						1 (i,iii)	1 to 1	424.1	3 (i,iii)	1 to 7	579.1
Community & Hospital	517						3 (ii,iii)	2 to 4	1178.6	10 (ii,iii)	4 to 25	970.8
*p values*^*a*^							*<0*.*001*			*<0*.*001*		
**Age at death (years)**												
<50	128	24.2	21.9	23.4	30.5	*	2 (i)	1 to 4	910.2	8	2 to 27	864.8
50–59	248	26.2	23.8	23.0	27.0	*	2	1 to 3	789.8	7	2 to 20	822.7
60–69	566	30.4	25.8	20.5	23.3	*	2	1 to 3	802.1	6	2 to 19.25	806.2
70–79	803	36.0	25.2	18.9	19.9	n/s	2	1 to 3	819.4	6	2 to 17	788.2
80+	729	43.9	23.6	16.2	16.3	*	2 (i)	1 to 3	748.2	6	2 to 16.5	779.4
*p values*^*a*^		*[—————————-<0*.*001—————————-]*				*<0*.*001*	*0*.*012*			*0*.*465*		
**Gender**												
Male	1311	38	25.5	17.2	19.4	*	2	1 to 3	789	6	2 to 16	782
Female	1163	32.6	23.5	21.3	22.6	*	2	1 to 3	809.3	6	2 to 21	816.7
*p values*^*a*^		*[——————————0*.*002——————————]*				*<0*.*01*	*0*.*356*			*0*.*132*		
**IMD deprivation quintile**												
Quintile 1—Most deprived	773	35.1	23.4	17.9	23.7	n/a	2	1 to 3	809	6	2 to 20	801.8
Quintile 2	480	34.4	24.8	19.2	21.7	n/a	2	1 to 3	825.6	7	3 to 20	833.2
Quintile 3	393	35.9	25.2	21.4	17.6	n/a	2	1 to 3	786.4	7	2 to 19	800.8
Quintile 4	499	36.5	25.1	18.8	19.6	n/a	2	1 to 3	775.2	6	2 to 17.5	795.6
Quintile 5—Least deprived	329	35.9	25.2	19.8	19.1	n/a	2	1 to 3	786.6	5	1 to 15	744.2
*p values*^*a*^		*[——————————0*.*728—————————-]*				*0*.*965*	*0*.*608*			*0*.*312*		
**At least one hospital admission at any point from first cancer diagnosis**												
Yes	1218	34.1	24.9	19.5	21.6	n/a	2	1 to 3	816	7	2 to 20	811.6
No	1256	36.8	24.2	18.8	20.2	n/a	2	1 to 3	781.8	6	2 to 17	786.3
*p values*^*a*^		*[——————————0*.*553——————————]*				*0*.*172*	*0*.*120*			*0*.*272*		

a = p values from Chi-square (categorical), Mann-Whitney (continuous, two comparison groups), or Kruskal-Wallis (continuous, three or more comparison groups) tests

* = post-hoc z-test p values less than 0.05 (after Bonferroni correction); IQR = Interquartile range; n/s = Not significant; n/a = Not applicable; i,ii,iii,iv indicates post-hoc comparison between pair of categories resulted in p value less than 0.05 (after Bonferroni correction)

Provision of palliative care was significantly related to age and sex (p<0.001 and p = 0.002 respectively). Patients less likely to receive palliative care were over 80 years of age and male. For patients who received palliative care, the number of palliative care events was significantly related to the patient’s age (p = 0.012), with patients aged under 50 years at death receiving significantly more palliative care events than patients aged 80 years or over (p = 0.011). There were no significant associations between the IMD deprivation quintiles for any of the palliative care provision outcomes assessed.

### Cancer characteristics

Palliative care provision was significantly associated with both cancer type and duration of illness (both p<0.001), Table 2. Comparing no palliative care to some palliative care showed that patients with upper gastrointestinal cancer were significantly more likely to receive palliative care, while patients with lung cancer were significantly less likely to receive any palliative care (p = 0.010 overall). For patients who received palliative care no significant associations were identified between cancer type, for both duration and intensity of palliative care.

**Table 2:** Type and duration of cancer by palliative care provision.

	Total (No.)	Palliative care received (Row %)				None vs. Any p value^*a*^	Sub-group receiving palliative care (n = 1597)					
		None	Community only	Hospital only	Community & Hospital		Palliative care events			Palliative care duration (weeks)		
							Median	IQR	Mean Rank	Median	IQR	Mean Rank
**Cancer diagnosis (first diagnosis)**												
Head and neck	111	37.8	19.8	29.7	12.6	n/s	1	1 to 2	672.3	5	1 to 13	725.8
Upper gastrointestinal	387	28.4	25.8	21.2	24.5	*	2	1 to 3	827.4	7	2 to 16	811.3
Colorectal	327	34.6	25.1	15.6	24.8	n/s	2	1 to 3	853	6	2 to 20	801.1
Lung	656	40.5	24.4	15.5	19.5	*	2	1 to 3	789.3	6	2 to 16	767.2
Breast	235	34.5	28.5	17.0	20.0	n/s	2	1 to 4	831.2	7	1 to 30.25	828.3
Gynaecological	150	29.3	19.3	28.7	22.7	n/s	1.5	1 to 3	740.3	5	2 to 22.25	778.6
Prostate	232	35.8	28.9	15.5	19.8	n/s	2	1 to 3	796.9	9	2 to 25.5	887.4
Urological	191	34.6	22.0	25.1	18.3	n/s	2	1 to 3	754.3	6	2 to 16	766.1
Central nervous system	59	45.8	32.2	11.9	10.2	n/s	2	1 to 4	839	10	5.25 to 19	926.1
All other cancer sites	126	35.7	15.1	24.6	24.6	n/s	2	1 to 3	786.5	6	2 to 19	775.8
*p values*^*a*^		*[—————————-<0*.*001—————————-]*				*0*.*01*	*0*.*102*			*0*.*144*		
**Duration of illness**												
0 to under 3 months	332	31.9	13.0	29.5	25.6	n/a	2 (i,ii)	1 to 3	706.9	3 (i,ii,iii,iv,v)	1 to 5	510
3 to under 6 months	292	41.1	20.5	16.1	22.3	n/a	2	1 to 3	789.2	7 (i,vi)	2 to 14	755.9
6 to under 9 months	241	33.6	29.0	14.9	22.4	n/a	2 (i)	1 to 3	865.3	7 (ii)	2 to 21	805
9 to under 12 months	215	31.6	28.8	24.2	15.3	n/a	1	1 to 3	718.4	7 (iii)	2 to 26	825
1 to under 2 years	496	36.7	25.8	17.5	20.0	n/a	2	1 to 3	804	8 (iv)	2 to 22.25	851.6
2 or more years	898	35.6	27.2	17.0	20.2	n/a	2 (ii)	1 to 3	837.4	9 (v,vi)	2 to 32.25	888
*p values*^*a*^		*[—————————-<0*.*001—————————-]*				*0*.*159*	*<0*.*001*			*<0*.*001 *		

a = p values from Chi-square (categorical), Mann-Whitney (continuous, two comparison groups), or Kruskal-Wallis (continuous, three or more comparison groups) tests

* = post-hoc z-test p values less than 0.05 (after Bonferroni correction); IQR = Interquartile range; n/s = Not significant; n/a = Not applicable; i,ii,iii,iv,v indicates post-hoc comparison between pair of categories resulted in p value less than 0.05 (after Bonferroni correction)

While duration of illness was not significantly associated with whether or not palliative care was received it was significantly associated with the type of palliative care received (p<0.001). The results suggest hospital only palliative care was more likely for patients with a short duration of illness while community palliative care was more likely for patients with a longer duration of illness. For those who received palliative care duration of illness was significantly associated with both duration of palliative care and number of palliative care events (both p<0.001). Patients dying within three months of first diagnosis received significantly fewer palliative care events compared with patients dying six to nine months after diagnosis (p = 0.007), and patients dying two or more years after diagnosis (p = 0.002). Patients who died within three months of diagnosis received palliative care for a significantly shorter period of time compared to all other illness durations (all p<0.001) (Table 2).

### Therapies received

Palliative care provision was significantly associated with receiving chemotherapy, radiotherapy or an opioid prescription within the last year of life (all p<0.001). For patients who received palliative care, the duration of palliative care was significantly longer for those who received any of the three therapies (all p<0.001). Patients who received at least one opioid prescription within the last year of life and patients who received radiotherapy received significantly more palliative care events, compared to patients who did not receive each of the therapies (p<0.001 and p = 0.035 respectively). (Table 3).

**Table 3:** Therapies received by palliative care provision.

	Total (No.)	Palliative care received (Row %)				None vs. Any p value^*a*^	Sub-group receiving palliative care (n = 1597)					
		None	Community only	Hospital only	Community & Hospital		Palliative care events			Palliative care duration (weeks)		
							Median	IQR	Mean Rank	Median	IQR	Mean Rank
**Opioid prescription within the last year of life**												
Yes	222	20.5	34.0	16.5	29.0	*	2	1 to 4	904.0	9	3 to 26	896.3
No	655	47.1	17.1	21.2	14.6	*	1	1 to 2	675.9	4	1 to 12	685.0
*p values*^*a*^		*[————————-<0*.*001—————————]*				*<0*.*001*	*<0*.*001*			*<0*.*001*		
**Chemotherapy received**												
Yes	414	29.0	27.3	20.2	23.5	*	2	1 to 3	812.3	7	2 to 21	829.1
No	463	44.3	20.8	17.6	17.3	*	2	1 to 3	775.8	5	2 to 14	746.6
*p values*^*a*^		*[————————-<0*.*001—————————]*				*<0*.*001*	*0*.*11*			*<0*.*001 *		
**Radiotherapy received**												
Yes	496	35.1	27.7	16.7	20.5	*	2	1 to 3	819.0	8	2 to 23	849.4
No	381	36.0	20.3	22.3	21.4	*	2	1 to 3	772.0	5	2 to 14	730.7
*p values*^*a*^		*[————————-<0*.*001—————————]*				*0*.*634*	*0*.*035*			*<0*.*001 *		

a = p values from Chi-square (categorical), Mann-Whitney (continuous, two comparison groups), or Kruskal-Wallis (continuous, three or more comparison groups) tests

* = post-hoc z-test p values less than 0.05 (after Bonferroni correction); IQR = Interquartile range; n/a = Not applicable

### Multinomial multivariable logistic modelling

The results from the multinomial multivariable logistic regression model, presented in Table 4, show that the type of palliative care received was significantly associated with age, gender, first cancer diagnosis and duration of illness (see S2 Table for uni-variable logistic regression).

**Table 4:** Odds ratios (95% confidence intervals) from multinomial multivariable logistic regression comparing sources of palliative care, compared with no palliative care, by patient characteristics.

		Multinomial regression (Reference = No palliative care)			
Patient characteristics		Community only	Hospital only	Community and Hospital	Overall p value^a^
**Demographic characteristics**					
	**Age at death (years) **				
	<50	1.08 (0.60–1.97)	*2.12 (1.18–3.84)	*2.55 (1.43–4.54)	<0.005
	50–59	1.24 (0.80–1.93)	*1.96 (1.25–3.07)	*2.23 (1.43–3.49)	
	60–69	1.21 (0.87–1.68)	*1.66 (1.17–2.35)	*1.75 (1.24–2.48)	
	70–79	1.14 (0.86–1.51)	*1.37 (1.01–1.86)	1.35 (0.99–1.83)	
	80+ (REFERENCE)	1	1	1	
	**Gender **				
	Male	0.88 (0.68–1.14)	*0.66 (0.50–0.86)	*0.66 (0.50–0.87)	<0.005
	Female (REFERENCE)	1	1	1	
	**IMD deprivation quintile **				
	Quintile 1—Top 20% most deprived	1.00 (0.69–1.44)	0.94 (0.64–1.38)	1.32 (0.90–1.95)	0.733
	Quintile 2	1.18 (0.79–1.74)	1.10 (0.73–1.67)	1.36 (0.90–2.07)	
	Quintile 3	1.24 (0.83–1.85)	1.20 (0.79–1.84)	1.13 (0.73–1.77)	
	Quintile 4	1.04 (0.71–1.53)	1.03 (0.69–1.55)	1.14 (0.75–1.72)	
	Quintile 5—Top 20% most affluent (REFERENCE)	1	1	1	
	**At least one hospital admission at any point from first cancer diagnosis**				
	Yes	0.95 (0.75–1.20)	1.01 (0.79–1.29)	1.05 (0.82–1.34)	0.902
	No (REFERENCE)	1	1	1	
**Cancer characteristics**					
	**First diagnosis cancer site**				
	Head and neck	0.90 (0.49–1.63)	*2.57 (1.48–4.44)	0.81 (0.41–1.60)	<0.001
	Upper gastrointestinal	*1.76 (1.20–2.58)	*1.87 (1.24–2.80)	*2.03 (1.36–3.02)	
	Colorectal	1.33 (0.89–1.97)	1.32 (0.85–2.06)	*1.91 (1.27–2.88)	
	Trachea, bronchus and lung (REFERENCE)	1	1	1	
	Breast	1.13 (0.71–1.80)	1.04 (0.62–1.75)	0.97 (0.59–1.61)	
	Gynaecological	1.12 (0.63–1.97)	*2.18 (1.27–3.74)	1.57 (0.89–2.77)	
	Prostate	1.40 (0.88–2.20)	1.61 (0.95–2.70)	*1.89 (1.15–3.12)	
	Urological	1.26 (0.79–2.01)	*2.33 (1.47–3.71)	1.61 (0.98–2.64)	
	Central nervous system	1.31 (0.66–2.57)	0.66 (0.27–1.61)	0.52 (0.20–1.35)	
	All other cancer sites	0.70 (0.38–1.28)	1.46 (0.85–2.51)	1.33 (0.77–2.32)	
	**Duration of illness**				
	0 to under 3 months	0.87 (0.56–1.35)	*2.76 (1.88–4.06)	*2.37 (1.58–3.53)	<0.001
	3 to under 6 months	0.80 (0.54–1.19)	0.97 (0.64–1.47)	1.21 (0.81–1.81)	
	6 to under 9 months	1.29 (0.87–1.92)	1.02 (0.65–1.62)	1.37 (0.89–2.10)	
	9 to under 12 months	1.36 (0.90–2.05)	*1.65 (1.08–2.54)	0.95 (0.58–1.54)	
	1 to under 2 years	0.98 (0.72–1.33)	1.01 (0.72–1.41)	1.00 (0.72–1.39)	
	2 or more years (REFERENCE)	1	1	1	
**Therapies received**					
	**Opioid prescription within the last year of life **				
	Yes	*4.38 (3.49–5.50)	*1.82 (1.42–2.34)	*4.34 (3.41–5.52)	<0.001
	No (REFERENCE)	1	1	1	
	**Chemotherapy received **				
	Yes	*1.63 (1.27–2.10)	*1.69 (1.29–2.21)	*1.63 (1.25–2.14)	<0.001
	No (REFERENCE)	1	1	1	
	**Radiotherapy received**				
	Yes	*1.49 (1.16–1.90)	0.92 (0.71–1.18)	1.22 (0.94–1.57)	<0.005
	No (REFERENCE)	1	1	1	

a = p value from the likelihood ratio test based on Chi-square statistics

* = Significant at the 5% level (2-tailed)

Significant associations were also found between receiving palliative care and receiving opioids or cancer therapies. The odds of receiving palliative care (community only, hospital only, or both community and hospital) compared with no palliative care were significantly greater for patients who received an opioid prescription within the last year of life (Community only: OR = 4.38, 95% CI = 3.49–5.50; Hospital only: OR = 1.82, 95% CI = 1.42–2.34; Community and Hospital: OR = 4.34, 95% CI = 3.41–4.52) or who received chemotherapy (Community only: OR = 1.63, 95% CI = 1.27–2.10; Hospital only: OR = 1.69, 95% CI = 1.29–2.21; Community and Hospital: OR = 1.63, 95% CI = 1.25–2.14).

The odds of receiving community only palliative care, compared with no palliative care was also significantly greater for patients who received radiotherapy (OR:1.49, 95% CI:1.16–1.90), however there was no significant difference between patients who did or did not received radiotherapy in the odds of receiving hospital only or hospital and community palliative care compared with no palliative care.

Overall the sub group analysis (see S3 Table), reflect the overall results. For chemotherapy, however, the subgroup analysis showed that patients who died within six months of diagnosis had significantly lower odds of receiving hospital palliative care if they received chemotherapy (OR = 0.56, 95% CI = 0.33–0.97) or radiotherapy (OR = 0.40, 95% CI = 0.24–0.68). This suggests that duration of illness may be a moderator in the relationship between receiving chemotherapy or radiotherapy and receiving palliative care, that is, patients diagnosed late may be less likely to receive chemotherapy or radiotherapy and hospital palliative care.

## Discussion

Two thirds of cancer patients in our study received some form of palliative care before death. This proportion is consistent with other studies undertaken in the US and Canada [12–14]. Lack of engagement with palliative care is not necessarily synonymous with unmet need. A study exploring carers’ insights into palliative care involvement among cancer patients found lack of engagement may relate to individual preference or lack of a perceived need by the cancer patient [12]. Distinguishing such patients from those who would welcome and benefit from palliative care is reliant on effective clinical assessment and communication and the opportunity to revisit discussions about palliative care throughout the cancer journey.

Our finding that patients over 80 years of age were less likely to receive palliative care is consistent with the existing understanding that in the UK only 16 per cent of patients receiving specialist palliative care are aged 85 or over, although 39 per cent of deaths occur in this age group [15] and previous evidence showing that younger cancer patients were more likely to access inpatient hospice services [16]. Whether this implies younger people have greater support needs or are more effective in accessing support is unclear but nevertheless this suggests older patients in particular may benefit from more proactive assessment.

Overall receiving chemotherapy, radiotherapy or an opioid were the most important factors associated with receiving community palliative care, and important factors associated with receiving hospital palliative care, though duration of illness may play a role in the likelihood of receiving these services. Being in regular contact with oncology services may therefore facilitate access to palliative care, particularly for those diagnosed at least six months before death. This finding may also contribute to our understanding about why having a cancer diagnosis has traditionally been, and continues to be, the main determinant of access to specialist palliative care services [16]. This finding suggests that efforts to improve access to palliative care should focus on those patients who have never received chemotherapy or radiotherapy or whose treatment has ended.

Patients who had received a prescription for an opioid were also more likely to access palliative care, though it is not clear whether the opioid prescription drives the palliative care referral or that palliative care improves access to an opioid analgesic. A multicentre study of 1450 patients with cancer pain comparing oncology care alone with early integration of palliative care alongside oncology care found that early access to palliative care was associated with a 31% reduced risk of suffering from severe pain [17] which supports the hypothesis that palliative care could be a mechanism through which to access opioid analgesia. In a recent study we found that access to an opioid was also related to age and cancer diagnosis, possibly suggesting that other mediators or moderator factors may explain this association [18]. Further research is needed to fully understand the nature of the association between opioid prescriptions and palliative care.

The average duration of palliative care involvement in our study was six weeks and the average number of contacts was two. This is a relatively short period of time and a surprisingly low dose intensity in relation to the level of engagement the US research evidence suggests is optimal [1–5]. The interventions in the US trials varied but common characteristics were an assessment and several follow up consultations over a period of 3–6 months. We found that the duration of hospital palliative care was even shorter and provided on fewer occasions than community palliative care. A greater understanding is needed about the outcomes associated with the current level of palliative care input to establish which benefits would be derived from earlier more intensive involvement.

This study has limitations. First, the population is derived from a single UK city, and although we have been able to determine the population is broadly representative of the UK cancer population in terms of prevalence of cancer type, age, sex, and survival, the extent to which palliative care involvement is representative of national activity is harder to determine. Leeds has two large hospices and a hospital palliative care team so is relatively well provisioned in terms of palliative care and the level of access reported here may consequently be higher than elsewhere in the UK. The data used in this study are derived from a live clinical system and as such are likely to represent errors or omissions inherent within that system. We also acknowledge that we exercised caution in selecting the read codes to identify community palliative care involvement and may mean the extent of community palliative care involvement could be higher than reported here.

## Conclusion

The study provides much needed evidence to support the implementation of the enhanced supportive care initiative across the UK National Health Service. We have for the first time reported duration of palliative care and intensity of palliative care input in relation to cancer patient characteristics and context of palliative care within the UK NHS. Timely supportive care for all cancer patients is advocated but results from this study suggest that older patients and those who do not receive cancer therapies miss out. These patients should be targeted for screening to identify palliative care needs. This information enables benchmarking of UK practice against the international research evidence and identify which patients are currently under represented in terms of access to palliative care.

## Supporting information

S1 Table Read codes extracted from SystmOne. (DOCX)

S2 Table Odds ratios (95% confidence intervals) from multinomial uni-variable logistic regression comparing sources of palliative care, compared with no palliative care, by patient characteristics. (DOCX)

S3 Table Subgroup analysis odds ratios (95% confidence intervals) from multinomial multivariable logistic regression comparing sources of palliative care, compared with no palliative care, by therapies received stratified by survival time. (DOCX)
