# Supplementary material for: Access to hospital and community palliative care for patients with advanced cancer: A longitudinal population analysis
Source: PLoS One. 2018 Aug 8;13(8):e0200071. doi: 10.1371/journal.pone.0200071 (PMC6082504; doi:10.1371/journal.pone.0200071)
Supplement: S1 Table — (DOCX) [file pone.0200071.s001.docx]

Table S1. Read codes extracted from SystmOne

| **Read codes** | **Indicate active palliative care?** |
| --- | --- |
| (1Z01.) Terminal illness - late stage | No |
| (8BA2.) End of life care | No |
| (8H6A.) Refer to terminal care consult | Yes |
| (8H7L.) Refer for terminal care | Yes |
| (9EB5.) DS 1500 Disability living allowance completed | No |
| (Xa9tS) For resuscitation | No |
| (Xa9tT) Not for resuscitation | No |
| (XaAex) Referral to palliative care service | Yes |
| (XaAg6) Referral to palliative care physician | Yes |
| (XaAPW) Under care of palliative care physician | Yes |
| (XaAT5) Seen by palliative care physician | Yes |
| (XaAWN) Seen by palliative care medicine - service | Yes |
| (XaEJE) Palliative care | No |
| (XaIlk) Referred to community specialist palliative care team | Yes |
| (XaIpI) Palliative treatment | Yes |
| (XaIpl) Final days pathway | No |
| (XaIpX) Preferred place of death | No |
| (XaIse) Specialist palliative care treatment | Yes |
| (XaIsy) Preferred place of death discussed with patient | No |
| (XaIt6) Specialist palliative care treatment - daycare | Yes |
| (XaIt7) Specialist palliative care treatment - outpatient | Yes |
| (XaJ3g) Preferred place of death: home | No |
| (XaJ3h) Preferred place of death: hospice | No |
| (XaJ3j) Preferred place of death: hospital | No |
| (XaJ3k) Preferred place of death: nursing home | No |
| (XaJv2) On gold standards palliative care framework | No |
| (XaLwc) Resuscitation discussed with patient | No |
| (XaLwd) Resuscitation discussed with carer | No |
| (XaMhi) Liverpool care pathway for the dying | No |
| (XaPmq) Issue of palliative care anticipatory medication box | No |
| (XaQ8S) Anticipatory palliative care | No |
| (XaQg1) Last days of life | No |
| (XaQzq) Preferred place of death: pt unable to express preference | No |
| (XaQzr) Preferred place of death: discussion not appropriate | No |
| (XaQzt) Preferred place of death: patient undecided | No |
| (XaR50) GSF supportive care stage 1 - advancing disease | No |
| (XaR53) GSF supportive care stage 2 - increasing decline | No |
| (XaR5A) GSF supportv care stge 3 - last days: cat C - wks prognosis | No |
| (XaRFF) Has end of life advance care plan | No |
| (XaRFG) On end of life care register | No |
| (XaX46) GSF supportv care stge 3 - last days: cat B - mth prognosis | No |
| (ZV57C) [V]Palliative care | No |
