# Supplementary material for: Access to hospital and community palliative care for patients with advanced cancer: A longitudinal population analysis
Source: PLoS One. 2018 Aug 8;13(8):e0200071. doi: 10.1371/journal.pone.0200071 (PMC6082504; doi:10.1371/journal.pone.0200071)
Supplement: S2 Table — (DOCX) [file pone.0200071.s002.docx]

Table S2. Odds ratios (95% confidence intervals) from multinomial uni-variable logistic regression comparing sources of palliative care, compared with no palliative care, by patient characteristics

|  | | Multinomial regression (Reference= No palliative care) | | | |
| --- | --- | --- | --- | --- | --- |
| Patient characteristics | | Community only | Hospital only | Community and Hospital | Overall p value^a^ |
| **Demographic characteristics** | | | | | |
|  | **Age at death (years)** | | | | |
|  | <50 | 1.68 (0.98-2.89) | *2.62 (1.52-4.52) | *3.38 (2.02-5.67) | <0.001 |
|  | 50-59 | *1.69 (1.13-2.52) | *2.38 (1.57-3.60) | *2.77 (1.86-4.14) |  |
|  | 60-69 | *1.58 (1.18-2.11) | *1.83 (1.33-2.51) | *2.06 (1.52-2.81) |  |
|  | 70-79 | *1.30 (1.01-1.68) | *1.43 (1.07-1.90) | *1.49 (1.12-1.98) |  |
|  | 80+ (REFERENCE) | 1 | 1 | 1 |  |
|  | **Gender** | | | | |
|  | Male | 0.93 (0.76-1.15) | *0.69 (0.55-0.86) | *0.74 (0.59-0.91) | <0.005 |
|  | Female (REFERENCE) | 1 | 1 | 1 |  |
|  | **IMD deprivation quintile** | | | | |
|  | Quintile 1 - Top 20% most deprived | 0.95 (0.68-1.33) | 0.92 (0.64-1.33) | 1.27 (0.88-1.81) | 0.728 |
|  | Quintile 2 | 1.03 (0.71-1.48) | 1.01 (0.68-1.50) | 1.18 (0.80-1.75) |  |
|  | Quintile 3 | 1.00 (0.68-1.46) | 1.08 (0.72-1.62) | 0.92 (0.60-1.40) |  |
|  | Quintile 4 | 0.98 (0.68-1.40) | 0.94 (0.63-1.39) | 1.01 (0.68-1.49) |  |
|  | Quintile 5 - Top 20% most affluent (REFERENCE) | 1 | 1 | 1 |  |
|  | **At least one hospital admission at any point from first cancer diagnosis** | | | | |
|  | Yes | 1.11 (0.90-1.37) | 1.12 (0.89-1.40) | 1.15 (0.93-1.43) | 0.553 |
|  | No (REFERENCE) | 1 | 1 | 1 |  |
| **Cancer characteristics** | | | | | |
|  | **First diagnosis cancer site** | | | | |
|  | Head and neck | 0.87 (0.50-1.51) | *2.05 (1.23-3.41) | 0.69 (0.37-1.31) | <0.001 |
|  | Upper gastrointestinal | *1.51 (1.01-2.11) | *1.94 (1.35-2.80) | *1.80 (1.27-2.54) |  |
|  | Colorectal | 1.21 (0.85-1.70) | 1.18 (0.79-1.76) | *1.49 (1.05-2.12) |  |
|  | Trachea, bronchus and lung (REFERENCE) | 1 | 1 | 1 |  |
|  | Breast | 1.38 (0.94-2.01) | 1.29 (0.83-2.00) | 1.21 (0.80-1.83) |  |
|  | Gynaecological | 1.10 (0.66-1.82) | *2.55 (1.58-4.11) | 1.61 (0.98-2.63) |  |
|  | Prostate | 1.34 (0.92-1.96) | 1.13 (0.72-1.78) | 1.15 (0.76-1.75) |  |
|  | Urological | 1.06 (0.69-1.63) | *1.90 (1.23-2.93) | 1.10 (0.70-1.75) |  |
|  | Central nervous system | 1.07 (0.63-2.17) | 0.68 (0.29-1.60) | 0.46 (0.19-1.15) |  |
|  | All other cancer sites | 0.70 (0.40-1.24) | *1.80 (1.08-3.00) | 1.43 (0.87-2.37) |  |
|  | **Duration of illness** | | | | |
|  | 0 to under 3 months | *0.53 (0.36-0.79) | *1.93 (1.38-2.07) | *1.42 (1.01-2.00) | <0.001 |
|  | 3 to under 6 months | *0.66 (0.46-0.93) | 0.82 (0.56-1.21) | 0.96 (0.67-1.36) |  |
|  | 6 to under 9 months | 1.13 (0.79-1.63) | 0.93 (0.60-1.44) | 1.18 (0.80-1.74) |  |
|  | 9 to under 12 months | 1.20 (0.82-1.75) | *1.60 (1.06-2.41) | 0.86 (0.55-1.35) |  |
|  | 1 to under 2 years | 0.92 (0.70-1.22) | 1.00 (0.73-1.38) | 0.96 (0.71-1.31 |  |
|  | 2 or more years (REFERENCE) | 1 | 1 | 1 |  |
| **Therapies received** | | | | | |
|  | **Opioid prescription within the last year of life** | | | | |
|  | Yes | *4.57 (3.66-5.72) | *1.80 (1.41-2.28) | *4.56 (3.62-5.76) | <0.001 |
|  | No (REFERENCE) | 1 | 1 | 1 |  |
|  | **Chemotherapy received** | | | | |
|  | Yes | *2.01 (1.63-2.49) | *1.76 (1.40-2.21) | *2.08 (1.66-2.60) | <0.001 |
|  | No (REFERENCE) | 1 | 1 | 1 |  |
|  | **Radiotherapy received** | | | | |
|  | Yes | *1.40 (1.13-1.73) | 0.77 (0.62-0.97) | 0.98 (0.79-1.22) | <0.001 |
|  | No (REFERENCE) | 1 | 1 | 1 |  |
| *a = p value from the likelihood ratio test based on Chi-square statistics; * = Significant at the 5% level (2-tailed)* | | | | | |
