# Supplementary material for: Access to hospital and community palliative care for patients with advanced cancer: A longitudinal population analysis
Source: PLoS One. 2018 Aug 8;13(8):e0200071. doi: 10.1371/journal.pone.0200071 (PMC6082504; doi:10.1371/journal.pone.0200071)
Supplement: S3 Table — (DOCX) [file pone.0200071.s003.docx]

Table S3. Subgroup analysis odds ratios (95% confidence intervals) from multinomial multivariable logistic regression comparing sources of palliative care, compared with no palliative care, by therapies received stratified by survival time^a^

|  | | Multinomial regression (Reference= No palliative care) | | | |
| --- | --- | --- | --- | --- | --- |
| **Therapies received** | | Community only | Hospital only | Community and Hospital | Overall p value^b^ |
| **Survival under six months from first diagnosis** | | | | | |
|  | **Opioid prescription within the last year of life** | | | | |
|  | Yes | *4.74 (2.78-8.11) | *2.47 (1.48-4.11) | *5.81 (3.55-9.53) | <0.001 |
|  | No (REFERENCE) | 1 | 1 | 1 |  |
|  | **Chemotherapy received** | | | | |
|  | Yes | 1.07 (0.59-1.93) | *0.56 (0.33-0.97) | 0.60 (0.34-1.04) | 0.059 |
|  | No (REFERENCE) | 1 | 1 | 1 |  |
|  | **Radiotherapy received** | | | | |
|  | Yes | 1.32 (0.74-2.36) | *0.40 (0.24-0.68) | 0.63 (0.37-1.06 | <0.001 |
|  | No (REFERENCE) | 1 | 1 | 1 |  |
| **Survival six months to two years from first diagnosis** | | | | | |
|  | **Opioid prescription within the last year of life** | | | | |
|  | Yes | *4.49 (3.12-6.47) | 1.28 (0.84-1.95) | *4.57 (3.05-6.85) | <0.001 |
|  | No (REFERENCE) | 1 | 1 | 1 |  |
|  | **Chemotherapy received** | | | | |
|  | Yes | *2.07 (1.38-3.10) | *2.35 (1.50-3.66) | *1.81 (1.14-2.87) | <0.001 |
|  | No (REFERENCE) | 1 | 1 | 1 |  |
|  | **Radiotherapy received** | | | | |
|  | Yes | *1.74 (1.16-2.60) | 0.91 (0.59-1.40) | 1.25 (0.80-1.93) | 0.019 |
|  | No (REFERENCE) | 1 | 1 | 1 |  |
| **Survival over two years from first diagnosis** | | | | | |
|  | **Opioid prescription within the last year of life** | | | | |
|  | Yes | *4.17 (2.89-6.02) | *2.08 (1.37-3.17) | *3.75 (2.50-5.64) | <0.001 |
|  | No (REFERENCE) | 1 | 1 | 1 |  |
|  | **Chemotherapy received** | | | | |
|  | Yes | *1.60 (1.06-2.40) | *1.99 (1.22-3.23) | *2.49 (1.54-4.05) | 0.001 |
|  | No (REFERENCE) | 1 | 1 | 1 |  |
|  | **Radiotherapy received** | | | | |
|  | Yes | 1.23 (0.83-1.82) | 1.04 (0.67-1.62) | 1.21 (0.78-1.86) | 0.699 |
|  | No (REFERENCE) | 1 | 1 | 1 |  |
| *Separate multinomial multivariable logistic regression models created for each stratified survival period, controlling for age, gender, IMD, hospital admission, and first diagnosis cancer site; b = p value from the likelihood ratio test based on Chi-square statistics; * = Significant at the 5% level (2-tailed)* | | | | | |
